# Watershed Infarct in Beta-Thalassemia Major Patient

**DOI:** 10.1155/2017/2736402

**Published:** 2017-03-27

**Authors:** Behnaz Ansari, Mohammad Saadatnia, Ali Asghar Okhovat

**Affiliations:** ^1^Isfahan Neurosciences Research Center, Alzahra Research Institute, Isfahan University of Medical Sciences, Isfahan, Iran; ^2^Department of Neurology, Isfahan University of Medical Sciences, Isfahan, Iran; ^3^Department of Neurology, Tehran University of Medical Sciences, Sina Hospital, Tehran, Iran

## Abstract

*Background*. The mechanism of stroke in beta-thalassemia was reported previously as cardioembolic and hypercoagulable state. However, there is no report of watershed infarct in beta-thalassemia anemia.* Method*. We present an adult *β*-thalassemia major patient with manifest asymptomatic chronic left carotid occlusion who suffered watershed infarct.* Result*. In the presence of asymptomatic chronic left internal carotid occlusion, we assumed that severe anemia (hemoglobin = 3) at admission leads to watershed infarct.* Conclusion*. Watershed infarct seems to be the cause of stroke in cases of *β*-thalassemia major with severe anemia. Blood transfusion can be applied in the setting of acute brain ischemia in such high risk patients.

## 1. Introduction

Thalassemia consists of inherited defects in the rate of synthesis of one or more of the globin chains of hemoglobin [1]. Beta-thalassemia is a condition of impaired production of beta globin chains, leading to relative excess of alpha globin chains [2]. Anemia, hemolysis, and ineffective erythropoiesis form the pathology for clinical presentations of beta-thalassemia [3–6]. Acute neurological complications in patients with beta-thalassemia have been reported such as cerebral ischemia, spinal cord fractures, and compression from extramedullary hematopoietic tumors [7–9].

Logothetis et al. reviewing 138 cases of beta-thalassemia major (B-TM) in Greece and described a stroke syndrome in 2 patients and transient ischemic attack in about 20% of the cases [10]. In Italian multicenter study of 735 patients with B-TM reported 16 thromboembolic events with presentation of headache, seizure, and hemiparesis [11]. In patients with B thalassemia/hemoglobin E disease and alpha thalassemia, cerebral thrombosis was detected [12, 13]. However, all these reports were from patients who were not given regular transfusions. The mechanism of stroke in beta-thalassemia was reported previously as hypercoagulable state and cardioembolic and large vessels thrombosis [14, 15]. However, watershed infarct in beta-thalassemia anemia has been rarely reported before.

We report a case of beta-thalassemia major with severe anemia that was not given regular transfusion and presented with stroke.

## 2. Case Report

**Figure 1:**
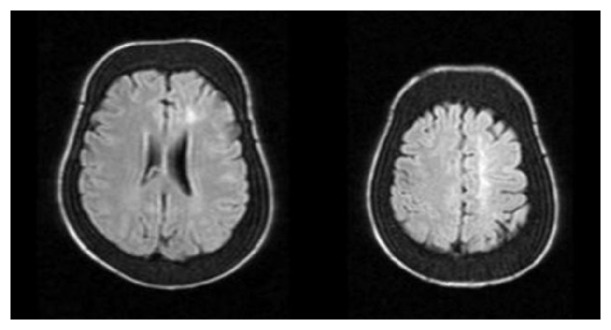
Magnetic resonance imaging in patient with thalassemia major showed flair signal intensity abnormalities in left watershed area and diploe space expansion.

**Figure 2:**
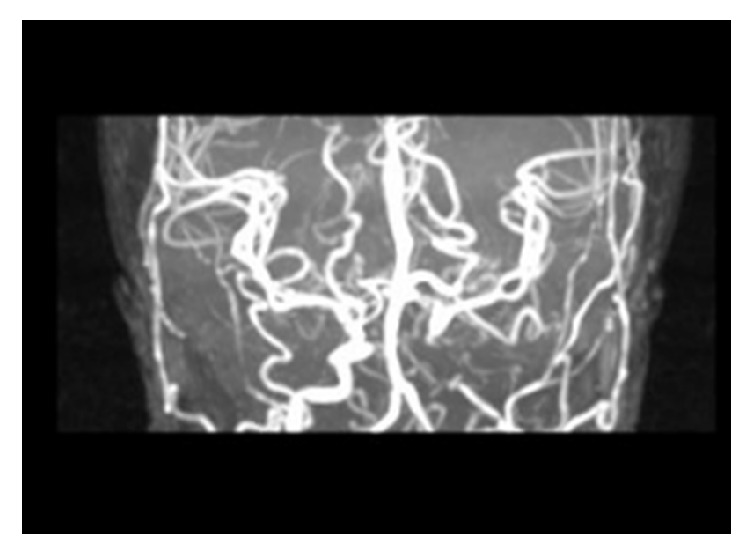
Magnetic resonance arteriography in patient with thalassemia major showed complete occlusion of left internal carotid and diffuse vasospasm.

In Nov 2014, a 25-year-old woman with past history of beta-thalassemia major was admitted to the hospital with right hemiparesis. She was a known case of beta-thalassemia major with regular blood transfusion until 9 years old. The mean hemoglobin of patient was 9 gr/dl. But she had twice hemolysis after transfusion, and after this situation, she refused to receive blood transfusion. She did not take any drug after this side effect. She had past history of gallbladder stone one month before the current hemiparesis and had undergone cholecystectomy. In this stage the mean Hb was 7 gr/dl, so before the surgery she received blood transfusion. In this admission, on general examination, she was undernourished with a short stature. Head and neck examination revealed depressed cranial vault, frontal bossing, retracted upper lip, and saddle nose (severe face deformity due to extramedullary hematopoiesis). On neurological examination, she was right hemiparesis (force: 3/5); her cranial nerves and sensory function were intact. In paraclinic tests, Hb was 3.4 gr/dl and echocardiography showed increased aortic flow. Abdominal sonography revealed hepatosplenomegaly. Brain MRI showed hypersignal intensity in left cortical watershed area (Figure 1). Brain MRA and CT angiography showed chronic total occlusion of left internal carotid artery at from C2 till C5 segment (Figure 2). In transcranial duplex study of cervical and cerebral vessels, internalization of left external carotid artery, reverse flow of left anterior cerebral artery, and high peak systolic velocities (PSV > 125 cm/s) in all transcranial vessels were shown. After blood transfusion without any thrombotic or anticoagulant drugs, hemiparesis was improved in three days.

Due to severe anemia after 3 months, she had undergone splenectomy and anemia was improved. Before surgery the mean Hb was 6 gr/dl and after that the mean Hb increased to 9 gr/dl and serum ferritin was 1500 mg/l. After 6 months of follow-up, modified Rankin scale was zero and transcranial duplex showed normal PSV.

## 3. Discussion

Watershed infarct is defined as an ischemic or blood flow blockage that is localized to the border zones [16]. Watershed strokes are localized to two primary regions of the brain and termed cortical watersheds (CWS) and internal watersheds (IWS) [17].

The causes of watershed infarct contain congestive heart failure, angiopathy, hypotension, hypertension, hyperlipidemia, carotid artery stenosis, and diseases such as sickle cell anemia [18–20].

Thalassemia is congenital hemolytic disorder caused by a partial or complete deficiency of alpha or beta globin chain synthesis. Ischemic strokes have been reported in 0.25% of patients with beta-thalassemia major [21].

In an Iranian study, stroke was documented in 0.46% of patients with beta-thalassemia major [22].

The presence of persistent hypercoagulable state combined with the infrequent thrombotic events suggests that thrombosis is largely a subclinical process in thalassemia [23, 24]. These thrombi could contribute to the pulmonary hypertension [25–30] and high frequency of ischemic brain lesions associated with asymptomatic (silent stroke) and symptomatic brain damage as detected by MRI [31]. Carotid artery intima media thickness (CIMT) shows a strong relationship with features of iron overload and atherosclerotic changes in beta-thalassemia major patients; however use of Doppler measurement of CIMT is recommend in beta-thalassemia major patients as a noninvasive diagnostic method to predict early atherosclerotic changes as well as in the follow-up to prevent progression of atherosclerosis [32].

Several etiologic factors play a role in hypercoagulable state in thalassemia [32–35].

The beneficial role of regular transfusions is illustrated by observation that thromboembolic accident is more frequently recorded with limited transfusion. Normal red blood cells (RBCs) can eliminate the abnormal aggregation observed with thalassemic RBCs [36].

In addition to asymptomatic and symptomatic stroke due to hypercoagulable state, cardioembolic and large vessels thrombosis also were reported as the cause of stroke in thalassemia [14, 15, 37, 38].

The relationship of iron overload effect on brain ischemia and infarction in beta-thalassemia major was evaluated in some articles and in southern Iran a higher frequency (66%) was reported for silent cerebral infarctions in transfusion-dependent patients with beta-thalassemia major [38]. But our case refused to receive transfusion due to hemolysis, so this theory was not accepted for this patient and on the other hand our patient has severe anemia and, after transfusion, hemiparesis was improved.

However, watershed infarct is rare type. The mechanism of watershed infarct in this patient is coexistence of chronic left internal carotid occlusion (ICA) and severe anemia. It seems left ICA occlusion alone, due to efficient collateral flow, could not lead to infarct; however superimposition of severe anemia leads to watershed infarct in the same side of ICA occlusion.

The etiology of ICA occlusion in this patient could be due to extramedullary hematopoiesis in sellar region, sphenoid bone, and petrous bone.

In conclusion, watershed infarct seems to be the cause of stroke in *β*-thalassemia major with severe anemia. Blood transfusion can be applied in the setting of acute brain ischemia in such high risk patients.
